# Supplementary material for: 3D Chemical Imaging by Fluorescence-detected Mid-Infrared Photothermal Fourier Light Field Microscopy
Source: Chem Biomed Imaging. 2023 Mar 20;1(3):260–7. doi: 10.1021/cbmi.3c00022 (PMC10302888; doi:10.1021/cbmi.3c00022)
Supplement: Supplementary file 1 — im3c00022_si_001.pdf [file im3c00022_si_001.pdf]

Supporting Information

3D Chemical Imaging by Fluorescence-detected Mid-Infrared Photothermal Fourier Light Field Microscopy

Danchen Jia‡, Yi Zhang‡, *, Qianwan Yang‡, Yujia Xue, Yuying Tan, Zhongyue Guo, Meng Zhang, Lei Tian* and Ji-Xin Cheng*

Email: zhangyi@bu.edu; leitian@bu.edu; jxcheng@bu.edu

1 Optical design of Fourier light field microscopy

The FMIP-FLF imaging system is designed based on a Fourier light field microscopy as seen in Figure 1b. To identify the subcellular structures, high spatial resolution is pursued in the FLF module. Since the lateral resolution of FLF microscopy is determined by $R_{xy} = \frac{\lambda N}{2NA}$, small occupancy ratio N of the lenslet array and large numerical aperture NA of the objective are desired. Consequently, we selected the lenslet array with high occupancy ratio N=2. Although oil objective or water objective provide higher NA, the strong FMIP background could overwhelm the FMIP signals of the samples. Thus, 100× objective with NA=0.95 is utilized to maximize the resolution.

- **Spatial resolution**

The lateral resolution of FLF microscopy is given as[1]

$$R_{xy} = \frac{\lambda N}{2NA} \qquad (S1)$$

Here, λ is the wavelength of emission (560 nm for fluorescence beads, P7220 PS-Speck™; 565 – 650 nm for Lipi-red, Dojindo). Consequently, the calculated lateral resolution is 580 nm when λ=560 nm. Meanwhile, the spatial resolution can also be constrained by the numerical aperture NA_{LA} of the lenslet array. In this case, $R_{xy} = \frac{\lambda}{2NA_{LA}} \times \frac{f_{FL}}{f_{LA}} \times \frac{1}{M}$, where the numerical aperture of the lenslet array NA_{LA} is 0.029, the focal length of the Fourier lens f_{FL} and the lenslet array f_{LA} is 150mm and 51.4 mm, the magnification of the objective M is 100. Thus, the effective NA of the lenslet array ($NA_{LA} \times \frac{f_{LA}}{f_{FL}} \times M = 0.97$) is larger than the objective, so the lateral resolution is still determined by NA of the objective.

The axial resolution of FLF microscopy is given as[1]

$$R_z = \frac{d_{LA} {R_{xy}}^2}{\lambda d_{max}} \qquad (S2)$$

Here, d_{LA} is the diameter of the lenslet, d_{max} is the distance from the outmost lenslet covered by the illumination beam to the center of the lenslet array.

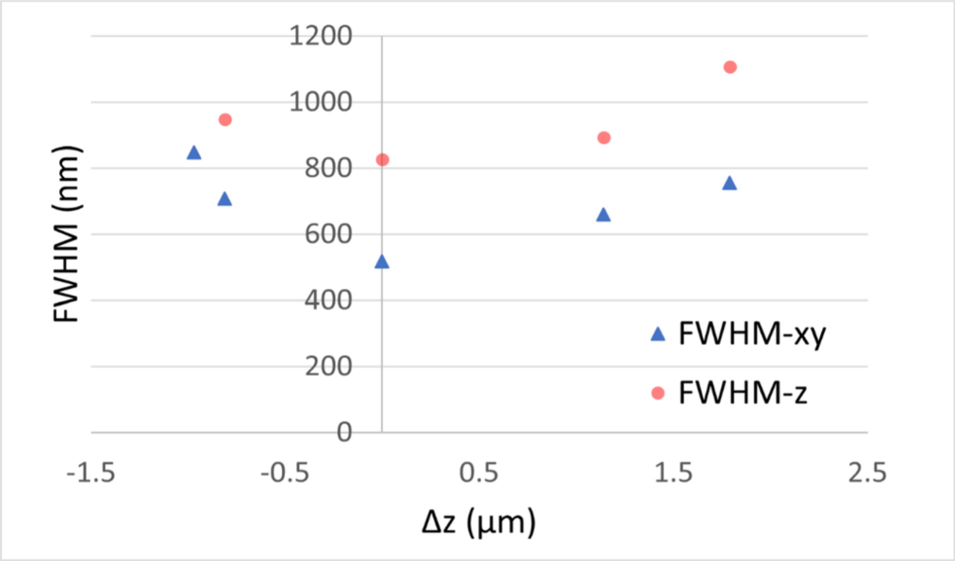

Figure S1. Full width at half-maximum of the lateral and axial cross section of 175 nm fluorescent beads at varying depths.

- **Field of view (FOV)**

The FOV of the FLF microscopy is determined by the image area after each micro-lens elements, given as $FOV = d_{LA} \times \frac{f_{FL}}{f_{LA}} \times \frac{1}{M}$. Consequently, the FOV is 87.5 μm theoretically. The FOV can be adjusted by an iris placed at the native image plane to avoid overlapping light field signals on the camera plane. Here, the MIR laser spot size is ~60×60 μm which determines the FOV of the FMIP-FLF system.

- **Depth of focus (DOF)**

The 3D reconstruction of the FLF microscopy is performed by the deconvolution between the light field measurement and the point-spread functions of the system. Thus, the DOF is determined by the range of detectable intensity considering the diffraction effect in the axial dimension. The DOF is calculated by two times of the axial FWHM of the PSF, $\frac{N^2\lambda}{NA^2} + \frac{N^2\lambda}{2S_r NA^2} = 5.7\mu m.$

- **Optimization of the optical design**

To further optimize the optical design of FMIP-FLF imaging system, the FOV can be improved without sacrificing the spatial resolution by redesigning the optical parameters of the FL and lenslet array with small camera pixel size. In order to meet the requirements of Nyquist sampling theory, the pixel size of the camera need to be smaller than $P = R_{xy} M \frac{1}{S_r} \frac{f_{LA}}{f_{FL}}$. For example, when the pixel size of the camera is 3.45 μm, f_{LA} = 50 mm, d_{LA} = 3 mm, f_{FL} =300 mm, the resulting FOV = 180 μm, DOF = 5.9 μm and R_{xy} = 585 nm, R_z = 850 nm.

In order to extend the application with longer imaging depth, such as neuron imaging, larger occupancy ratio N and lower NA is desired with an objective of low magnification which will sacrifice the spatial resolution. For example, a 20x objective with NA=0.5 can extend the DOF to 20 μm with R_{xy}=1 μm, R_z=2.8 μm and FOV of 416×416 μm with the proper optical design following the rationale described in the previous sections.

2 FMIP-FLF system calibration

FMIP-FLF reconstruction was performed by the deconvolution of 2D measurements with depth-dependent point-spread function (PSF) of the system. Here, the PSF is displayed with the depth-dependent color map calibrated experimentally (Experimental section). The axial range of measured PSFs determined the depth-of-focus we can restore with FMIP-FLF reconstruction.

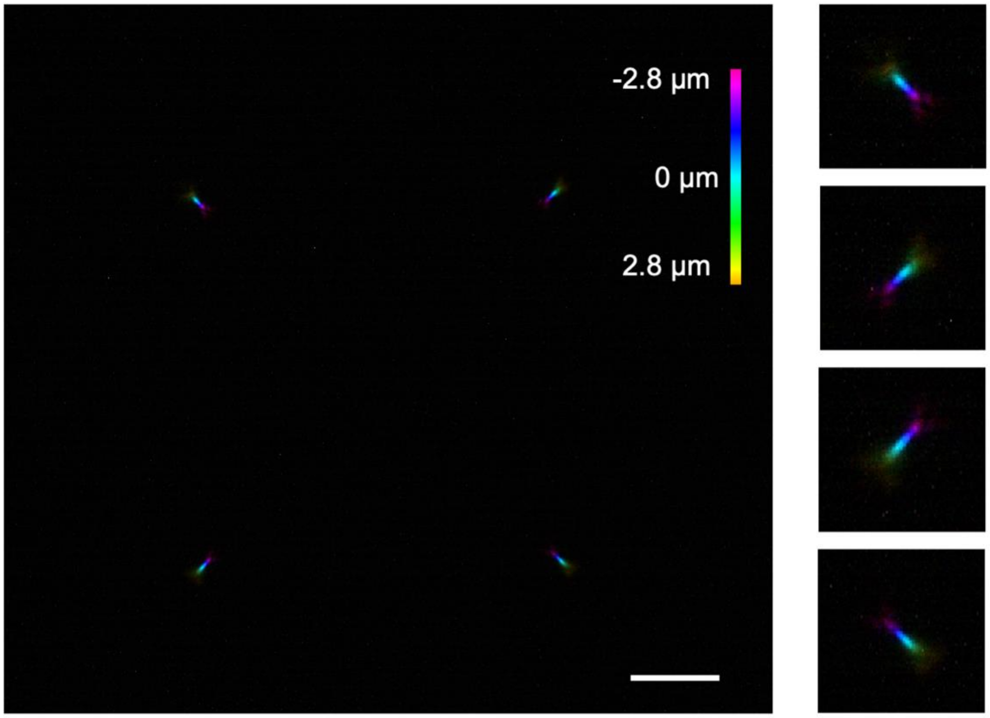

Figure S2. Point-spread function (PSF) of FLF microscopy measured from 175 nm fluorescent bead, insets: zoomed in PSF.

3 Fourier-transform infrared spectroscopy of fatty acids

Fourier-transform infrared spectroscopy (FTIR) of ^{13}C labeled fatty acid mixture showed a ~30 cm^{-1} peak shift to lower wavenumber compared with ^{12}C palmitic acid (major contents in the ^{13}C fatty acid mixture).

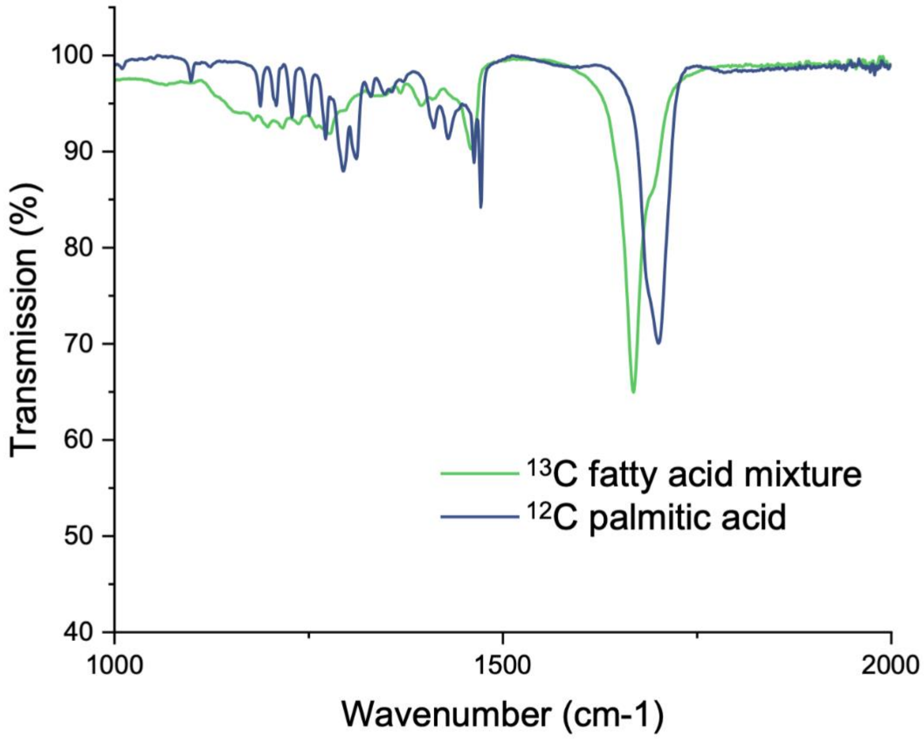

Figure S3. FTIR of ^{13}C fatty acid mixture (green) and palmitic acid (blue).

4 FMIP colormap of lipid contents in Mia Paca-2 and G3K cells

3D distribution of lipid contents in Mia Paca-2 cells ang G3K cells were demonstrated below. Here, green spots represent lipid droplets with FMIP peak at 1744 cm^{-1} ($^{12}C=O$), while red spots are lipid droplets with shifted FMIP peak at 1704 cm^{-1} ($^{13}C=O$). The following FMIP- FLF reconstruction stacks showed the chemical mapping of additional cells in Figure 4c, e.

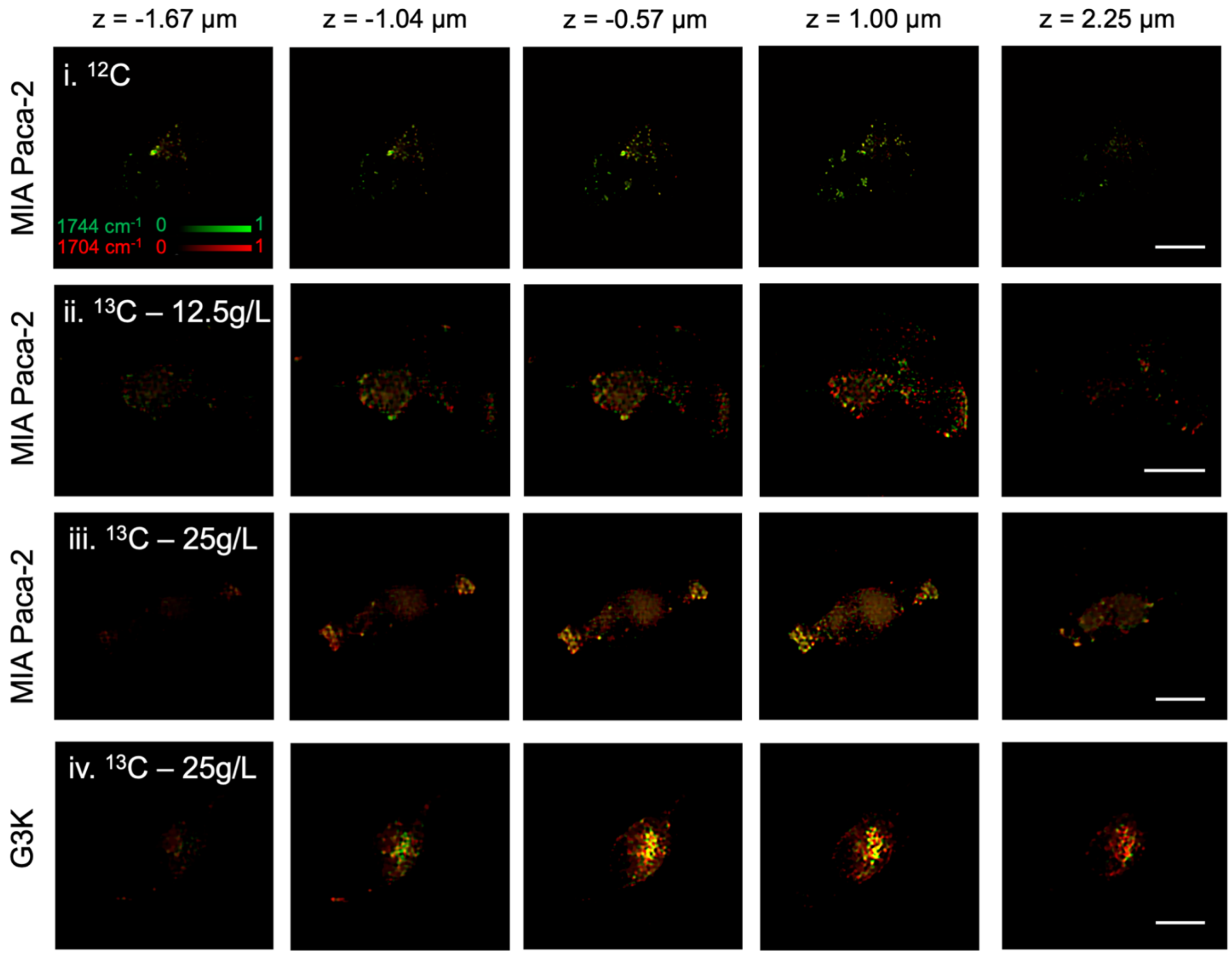

Figure S4. FMIP intensity colormap (red, 1704 cm^{-1}, green, 1744 cm^{-1}) at varying depths from 3D reconstructed stack of (i) MIA Paca-2 cells, (ii, iii) MIA Paca-2 cells treated with ^{13}C fatty acids of different concentration and (iv) G3K cells treated with ^{13}C fatty acids.

(1) Hua, X.; Liu, W.; Jia, S. High-resolution Fourier light-field microscopy for volumetric multi-color live-cell imaging. *Optica* **2021**, *8* (5), 614-620.
